# Natural flavonoids: Potential therapeutic strategies for non-alcoholic fatty liver disease

**DOI:** 10.3389/fphar.2022.1005312

**Published:** 2022-09-16

**Authors:** Panli Tan, Li Jin, Xiang Qin, Beihui He

**Affiliations:** ^1^ The First Affiliated Hospital of Zhejiang Chinese Medical University (Zhejiang Provincial Hospital of Traditional Chinese Medicine), Hangzhou, China; ^2^ School of Pharmaceutical Sciences, Zhejiang Chinese Medical University, Hangzhou, China

**Keywords:** non-alcoholic fatty liver disease, natural flavonoids, antioxidant, anti-inflammatory, intestinal flora, oxidat ive stress, inflammtion

## Abstract

The incidence of non-alcoholic fatty liver disease (NAFLD) is increasing rapidly worldwide; however, there are currently limited treatments for NAFLD. The disease spectrum includes simple fatty liver, non-alcoholic steatohepatitis (NASH), fibrosis, cirrhosis, and progression to hepatocellular carcinoma (NASH-HCC). The therapeutic effects of NAFLD remain controversial. Although researchers have conducted studies on the pathogenesis of NAFLD, its pathogenesis and anti-NAFLD mechanisms have not been fully elucidated. Previous studies have found that flavonoids, as natural substances with extensive pharmacological activity and good therapeutic effects, have excellent antioxidant, anti-inflammatory, metabolic disease improvement, anti-tumor, and other properties and can significantly alleviate NAFLD. Flavonoids could be further developed as therapeutic drugs for NAFLD. In this paper, the pathogenesis of NAFLD and the mechanisms of flavonoids against NAFLD are summarized to provide a theoretical basis for screening flavonoids against non-alcoholic liver injury.

## Introduction

Non-alcoholic fatty liver disease (NAFLD) is a chronic liver disease characterized by excessive fat deposition in hepatocytes, which is not caused by alcohol or other clear liver-damaging factors (Cobbina and Akhlaghi, 2017). The global incidence rate of NAFLD is approximately 25%, particularly in patients with diabetes and obesity (Mundi et al., 2020). NAFLD is the most common chronic liver disease worldwide and is expected to be the main cause of liver transplantation in the future (Younossi et al., 2016b). NAFLD encompasses a wide range of liver disorders, including simple fat accumulation in the liver cells, non-alcoholic steatohepatitis (NASH), fibrosis through the final stages of cirrhosis, and NASH-HCC (Cobbina and Akhlaghi, 2017). The incidence of NAFLD and NASH is related to sedentary lifestyle and excess dietary energy (Farrell et al., 2013). To date, the Food and Drug Administration has not approved any drugs for the treatment of NASH (Eduardo et al., 2015). Currently, NAFLD can be effectively alleviated only through non-drug management approaches, such as healthy lifestyle, diet, and moderate physical activity (Guillaume et al., 2015). Given the limited clinical treatment for NAFLD, the development of drugs that can effectively alleviate NAFLD is of great significance.

## Pathogenesis of non-alcoholic fatty liver disease

The pathogenesis of NAFLD remains unclear so far. However, recent studies have suggested a bidirectional association between NAFLD and metabolic syndrome, with type 2 diabetes increasing the risk of cirrhosis and related complications (Powell et al., 2021). Insulin resistance, diabetes mellitus, and genetic variations in transmembrane 6 superfamily member 2 (TM6SF2) and patatin-like phospholipase domain containing 3 (PNPLA3) play important roles in NAFLD progression (Cobbina and Akhlaghi, 2017). NAFLD is characterized by excessive fatty accumulation in the liver, while simple steatosis is considered pathologically benign. NASH generally indicates liver damage that can progress to severe pathology (Zhang et al., 2018).

The “two-hit” pathogenesis of NAFLD/NASH was widely accepted in the early stage (Chi, 2017). The “first hit” is characterized by an increase in hepatic fat, especially accumulation of hepatic triglycereides and insulin resistance. Once the accumulation of hepatic fat exceeds 5%, it corresponds to hepatic steatosis (Fang et al., 2018). The most direct cause of NAFLD is abnormal liver lipid metabolism, and a large quantity of free fatty acids and triglycerides that accumulate in liver cells (Xiaxia et al., 2019). The “second hit” is that reactive oxygen species (ROS) triggers an inflammatory cascade of liver parenchymal cells and fibrosis (Xiaxia et al., 2019). These effects include high levels of inflammatory cytokines, mitochondrial dysfunction, and oxidative stress. Necrotizing inflammation and fibrosis can progress and eventually lead to cirrhosis (Chi, 2017). However, the widely accepted theory is the “multiple-hit” pathogenesis (Ayonrinde et al., 2015). Changes due to the interaction of genetic and environmental factors, as well as the interactions between different organs and tissues, pancreas, gut, and liver, and broader metabolic dysfunction, are involved (Berardis and Sokal, 2014; Chi, 2017; Vlad et al., 2018). Moreover, scholars believe that environmental and genetic factors and the change in gut microbes in the induction of NAFLD in genetic predisposition, as well as intestinal flora changes lead to intestinal fatty acid, further activate the inflammatory pathways and release proinflammatory factors. Inflammatory cytokines increase liver inflammation and lipid accumulation, and the formation of gut-liver axis to a vicious cycle (Buzzetti et al., 2016; Xiaxia et al., 2019).

In recent years, the functional activity of key genes that synthesize proteins has been decisive in NAFLD. The PNPLA3 variant has been identified as the main genetic determinant of NAFLD. Variants with moderate effect sizes in TM6SF2, membrane bound O-acyltransferase domain containing 7 (MBOAT7), and glucokinase regulator (GCKR) were also shown to contribute significantly (Bellentani et al., 2004). PNPLA3, an enzyme that encodes I148M, is involved in the hydrolysis of triglycerides in adipocytes (Romeo et al., 2008). The lipid TM6SF2 is located in the endoplasmic reticulum and encodes E167K (rs58542926C/T), resulting in the loss of protein function, which in turn increases triglyceride deposition in the liver (Dongiovanni et al., 2015). Natural candidate genes are significantly involved in glucose and lipid metabolism during NAFLD development. Among the single nucleotide polymorphisms (SNPs) that lead to coding region mutations, such as PNPLA3 and TM6SF2, it is reasonable to infer that these defective proteins may be involved. For example, TM6SF2 mutants reduce liver production of very low-density lipoprotein (VLDL), thereby increasing the triglyceride (TG) content in the liver (Bonora et al., 2010).

Some studies have suggested that NAFLD progression follows the process of steatosis, lipotoxicity, and inflammation (Jou et al., 2008). The development of steatosis involves the interaction of many factors, such as dietary habits, gut flora, and genetic factors (Romeo et al., 2008; Jiang et al., 2015; Kirpich et al., 2015). Fat regenesis occurs through upregulation of adipogenic transcription factors, including sterol regulatory binding protein-1c (SREBP1c), carbohydrate-responsive element-binding protein (chREBP), and peroxisome proliferator-activated receptor gamma (PPAR-γ) (Anderson and Borlak, 2008). Fatty acids are mainly stored in the adipose tissue in the form of triacylglycerol. A previousstudy found that fatty acids in obese volunteers seemed to migrate from normal storage organs to the bone and liver tissue. Notably, FAT/CD36 (fatty acid translocation enzymes) promote fatty acid uptake by bone and liver tissues, which are significantly elevated in patients with obesity and NAFLD (Greco et al., 2008; Fabbrini et al., 2009). The accumulation of fat in the liver can lead to lipotoxicity and dysfunction of organelles, such as the mitochondria and endoplasmic reticulum (Browning and Horton, 2004; Bell et al., 2008). Steatosis further leads to the activation of IKKβ, which leads to increased signaling of the transcription factor nuclear factor kappa β (NF-κβ). Activation of NF-κβ induces the production of pro-inflammatory factors. These include tumour necrosis factor-alpha (TNF-α), interleukin 6 (IL-6), and interleukin-1beta (IL-1β) levels. These inflammatory factors can promote aggregation and activation of resident hepatic macrophages to further promote NASH inflammation (Ramadori and Armbrust, 2001; Fabbrini et al., 2009).

Oxidative stress may play an important role in NAFLD progression, and under normal physiological conditions, mitochondrial oxidation is the main oxidation pathway of fatty acid deposition. When ROS are overproduced during fatty acid oxidation, hydrogen polyunsaturated fatty acids are extracted from the liver, resulting in mass production of malondialdehyde (MDA) (Esterbauer et al., 1991). MDA can spread from its original site to other cells both inside and outside the cell, causing damage (Esterbauer et al., 1991). Catalase and glutathione levels decrease when ROS levels are elevated, and oxidative stress is exacerbated (Hongming et al., 2018). Lipid peroxidation increases collagen synthesis and cell death, which promotes steatosis and fibrosis (Huang et al., 2018).

Fatty acid outflow from the diet increases, and new fat formation releases free fatty acids from adipose tissue, contributing to TG accumulation in the liver, although to varying degrees (Yeh and Brunt, 2014). However, TG accumulation in the liver itself is not pathological, and may be protective in some cases. Hepatic diacylglycerol acyltransferase 2 (DGAT2) inactivation catalyzes TG synthase and reduces hepatic TG content but increases hepatitis and balloon-like changes (Brunt et al., 1999). This may seem paradoxical, but highlights the importance of liver fat in metabolic function. One possible mechanism for NASH-associated dysfunction involves a shift from minimal to substantial edema. This increase can be achieved by reducing the phosphatidylcholine (PC) levels (Machado et al., 2006) or lipid droplets coated with proteins (Soderberg et al., 2010; Angulo et al., 2013). Total PC levels were reduced in patients with both NAFLD and NASH (Ekstedt et al., 2006), which may be attributable to choline intake associated with NASH rather than choline deficiency (Richardson et al., 2007). In summary, NAFLD is a multifactorial disease with a complex pathogenesis. The prevention and treatment of NAFLD require further clinical and basic research.

## Classification of flavonoids

Some studies have confirmed that flavonoid intake is inversely related to the risk of NAFLD (Mm et al., 2019). The mechanisms by which flavonoids exert anti-NAFLD effects are mainly through ameliorating inflammation, oxidative stress, and lipid metabolism, and regulating intestinal microbiota imbalance and the related gut liver axis. Flavonoids are natural polyphenol compounds that exist widely in all types of natural plants. Now, more than 9,000 kinds of flavonoids have been identified with a structure of a two phenolic hydroxyl benzene ring (A- and B-loops) interconnected through the central three carbon atoms. The basic parent nucleus is called a 2- phenylchromone (Tsuji et al.), biosynthesis from acetic acid and phenylalanine in plants (Weston and Mathesius, 2013). Flavonoids can be divided into flavonoids, flavonols, orange ketones, isoflavones, anthocyanins, chalcones, and dihydrogen derivatives according to the difference in the three-carbon atomic structure of the linked A and B rings, such as whether the ring is formed, oxidized, or replaced (Tsuchiya, 2010). The types of flavonoids from different sources and their anti-NAFLD mechanisms of action are listed in Table 1.

**TABLE 1:** Flavonoids from several different sources and their anti-NAFLD mechanisms.

Class	Source of plant	Example	Mechanisms of anti-NAFLD	References
Flavone	Leaves, fruits, trunks of Asteraceae, Labiatae plants	Luteolin	Sirt1-AMPK signal pathway/Restoration of intestinal mucosal barrier damage and microbiota imbalance/Targeting the pro-inflammatory IL-1 and Il-18 pathways/Abolish lipid accumulation induced by LXR-SREBP-1c activation	Zhu et al. (2020) Xia et al. (2021) Abu-Elsaad and El-Karef, (2019) (Yin et al., 2017)
		Apigenin	Regulating hepatocyte lipid metabolism and oxidative stress/XO/NLRP3 pathways/PI3K/AKT-Dependent Activation/PPARγ/PGC-1α-Nrf2 pathway	Zhang et al. (2018b)
				Fan et al. (2017)
				Lv et al. (2019)
				Feng et al. (2017)
		Baicalein	Inhibited DNL and improved glucose tolerance, oxidative stress, liver histology, and hepatokine secretion/Via maintaining V-ATPase assembly/Reduce hepatic fat accumulation and to ameliorate NAFLD-related biochemical abnormalities	Sun et al. (2020)
				Zhu et al. (2019)
				Xing et al. (2021)
Flavonones	Citrus, Fabaceae, Moraceae, Myrtaceae	Eriodictyol	Induced a persistent increase in autophagic flux	Lascala et al. (2018) Geng et al. (2020)
		Hesperetin	PI3K/AKT-Nrf2-ARE pathway/Induction of GRP78 in hepatocytes	
				Li et al. (2021)
		Naringenin	down-regulating the NLRP3/NF-κB pathway	Ke et al. (2015)
			Enhancing Energy Expenditure and Regulating Autophagy via AMPK decreases adipose tissue mass and attenuates ovariectomy-associated metabolic disturbances	Yang et al. (2021)
				Chen et al. (2019b)
Flavonol	Leaves of various plants	Quercetin/Kaempferol	Ameliorating inflammation, oxidative stress, and lipid metabolism/Modulating intestinal microbiota imbalance and related gut-liver axis activation/IRE1a/XBP1s pathway	(Yin et al., 2017; Zhu et al., 2018)
				Yang et al. (2019)
		Galangin	Promoting Autophagy	Zhang et al. (2020)
		Myricetin	Regulating the expression of transcription factors of hepatic lipid metabolism, the antioxidant system, and pro-inflammatory cytokines	Choi et al. (2021)
			Modulating gut microbiota	Sun et al. (2021)
Isoflavone	Legumes	Daidzein	Direct regulation of hepatic *de novo* lipogenesis/Indirect control of adiposity and adipocytokines	Kim et al. (2011)
		Genistein	Directly targeted cyclooxygenase-1 activity as well as its downstream TXA2 biosynthesis/AMPK Activation	Zhong et al. (2017)
				Wang et al. (2018)
Anthocyanidin	Petals	Delphinidin	Induced endotoxemia and associated liver inflammation	Cremonini et al. (2022)
	Leaves Rhizomes	Malvidin	Nrf2/ARE Signaling Pathway/Hyperglycemia, insulin resistance, hyperlipidemia, and NAFLD in diabetic rats were alleviated	Zou et al. (2021)
				Xu et al. (2021)
Flavan-3OLS	Woody plants containing tannins	Catechin	GTE limitedly alters the hepatic metabolome/Reduce the contents of TG, TC, MDA, ALT and AST, increase the content of SOD	Gan et al. (2021)
				Sasaki et al. (2021)
		Galocatechin	Up-regulated mRNA and protein expressions of LPL, PPAR-α, CYP7A1 and CPT1, down-regulated PPAR-γ and C/EBP-α in liver of NAFLD mice	Liu et al. (2019)
		Theaflavin	Activating an AMPK Signaling Pathway by targeting Plasma Kallikrein/Anti-oxidant, anti-inflammatory, and anti-apoptotic mechanisms	Luo et al. (2012)
				Wenji Zhang et al. (2020)

## The main targets of flavonoids

**FIGURE 1:**
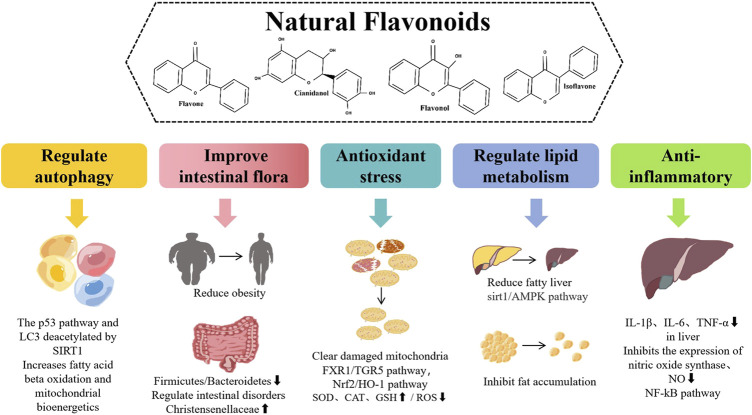
Different pharmacological effects and different mechanisms of natural flavonoids for alleviating NAFLD.

Flavonoids have a variety of pharmacological effects, including antitumor, antioxidant, antibacterial, antiviral, anti-inflammatory, and analgesic effects (Maleki et al., 2019; Makunga, 2020). Interestingly, flavonoids have positive effects on various NAFLD pathways, such as regulating lipid metabolism, insulin resistance, inflammation, and oxidative stress (Wier et al., 2015). Based on the above advantages, finding new anti-NAFLD drugs derived from plant flavonoids is a hot topic in current research (Figure 1).

### Improve the intestinal flora

Intestinal microbiota is involved in the pathogenesis of obesity, NAFLD, and metabolic syndrome (Abu-Shanab and Quigley, 2010). In NAFLD, changes in the gut microbiome and increased intestinal permeability lead to exposure of the liver to bacterial products from the gut, leading to chronic endotoxemia (Aron-Wisnewsky et al., 2013). Porras D found that quercetin could regulate intestinal microflora dysregulation in high fat diet (HFD)-induced NAFLD mice and reverse HFD-induced inhibition of short-chain fatty acids (SCFAs) production and related intestinal barrier dysfunction (Yin et al., 2017). Some scholars have pointed out through animal experiments that the use of flavonols can make mice intestinal *Firmicutes*/*Bacteroidetes* (F/B) ratio significantly reduced (Li, 2018). The F/B ratio is an indicator of intestinal health, and lowering it can reduce the risk of diabetes and obesity (Vebø et al., 2016). This suggests that flavonol protection of the intestinal flora can be achieved by reducing the F/B ratio. In addition, flavonol protection of the intestinal flora can also improve intestinal barrier function by increasing the expression of butyric acid receptors and conjunction in the intestinal mucosa (Chen et al., 2019). Anthocyanins can be digested by various intestinal structures to form metabolites that are transmitted throughout the body and exert positive biological effects (Aedín and Anne-Marie, 2017). Some studies have confirmed the results of *in vitro* microbial experiments. Anthocyanins can increase the growth rate of probiotics, such as *Lactobacillus acidophilus*or *Bifidobacterium*, and inhibit the growth of harmful bacteria, such as *Staphylococcus aureus* and *Salmonella typhimurium* (Hanju et al., 2018). Lima et al. (2019) confirmed through experimental studies that long-term supplementation of hesperidin and citra can effectively protect intestinal flora because the number and reproduction rate of *Bifidobacteria* and *Lactobacillus* in the intestinal tract are regulated by their influence, thus increasing the content of SCFAs to protect intestinal flora. Researchers studied the effects of flavonoids on intestinal microbes and found that when the dosage reached a certain concentration, it could significantly inhibit the reproduction of *Escherichia coli*, *Candida albicans*, *Staphylococcus aureus*, and *Bacillus* (Madheshwar and Perumal, 2017). Pure total flavonoids from citrus can regulate intestinal flora disorders, particularly Christensenellaceae, to attenuate NAFLD (He et al., 2021). Raw bowel tea polyphenols can reduce the level of *Firmicutes* in the feces of NAFLD mice, increase the minimum levels of *Bacteroidetes* and *Akkermansia*, and reduce the F/B ratio, acting as a regulator of the gut microbiome (Liu et al., 2019). Vine tea polyphenol reduced the F/B ratio and increased the relative abundance of *Akkermansia* in NAFLD mice (Xie et al., 2020).

Interactions between flavonoids and the microbiome contribute significantly to human health. The ability of flavonoids to regulate microbes also holds promise for dietary therapies that can be used to treat a variety of diseases associated with microbial disorders.

### Regulate lipid metabolism

Quercetin is widely distributed in photosynthetic plants, such as cereals, vegetables, fruit, tea leaves, and Chinese medicinal materials, and is the most abundant foodborne natural flavonoid (Martinon et al., 2002). Yang et al. (2019) established Type 2 diabetes mellitus (T2DM)-induced NAFLD and quercetin treatment models *in vivo* and *in vitro*, and found that quercetin reduced serum transaminase levels and significantly reduced liver histological changes. Wang (2021) found that mice fed a high-fat diet exhibited severe fat accumulation in their livers, and a large number of red fat droplets appeared in their visual field. After total flavonoids of Broussonetia papyrifera (TFBP) treatment, the fat content in the liver cells of mice decreased significantly and finally reached the levels observed in normal liver. These results indicated that TFBP had the ability toreduce fat accumulation in hepatocytes. Chian-jiunliou *et al.* staining with the fluorescent dye BODIPY 493/503 showed that incubating HepG2 cells with oleic acid-induced lipid accumulation and licorice chalcone significantly inhibited the aggregation of lipid droplets and confirmed that licorice chalcone promoted the Sirtuin1/AMP-activated protein kinase (Sirt1/AMPK) pathway in the liver *in vivo* and *in vitro*. It effectively inhibited adipogenesis and increased lipid decomposition and fatty acid β-oxidation in NAFLD mice (Liou et al., 2019). Luteolin, lycopene, and their combinations indirectly activate the SIRT1/AMPK pathway *in vivo* and *in vitro*, which in turn inhibits lipogenesis and increases β-oxidation, defending against the “two-hit” in NAFLD (Zhu et al., 2020).

### Antioxidant stress

Flavonoids may inhibit oxidative stress by regulating malondialdehyde (MDA), superoxide dismutase (SOD), and catalase (CAT). Wang (2021) found that total flavonoids from the leaves of *Broussonetia papyrifera* (TFBP) effectively inhibited the production of ROS, reduced the content of myeloperoxidase, improved the activity of SOD, and reduced injury to the body by oxidative stress. Western blot results showed that TFBP could regulate oxidative stress depending on the nuclear factor erythroid 2-related factor 2/heme oxygenase 1 (Nrf2/HO-1) signaling pathway, and promote Nrf2 entry into the nucleus of mouse liver cells and HO-1 production, thus improving the body’s ability to resist oxidative stress. Other researchers have concluded that theaflavins significantly reduce ROS production in steatotic hepatocytes and TNF-α production in LPS-stimulated RAW264.7 cells (Luo et al., 2012).

Cyanidin-3-O-glucoside is the most abundant anthocyanidin in the flavonoid family. Li *et al.* found that centaulin-3-O-glucoside eliminated damaged mitochondria to maintain mitochondrial homeostasis and alleviate oxidative stress (Yin et al., 2017). These results suggest that cybernin-3-O-glucoside alleviates NAFLD by activating PTEN-induced kinase 1 (PINK1)-mediated mitochondrial phagocytosis. In a NASH cell model, the levels of MDA and ROS were significantly increased significantly, while the levels of SOD, CAT, and GSH were significantly decreased. After stimulation with different concentrations of alpha-naphthoflavone (ANF), the level of SOD in the cells was decreased, but the level of SOD was significantly increased. Furthermore, MDA and ROS levels in the liver tissues of HFD-fed mice with different concentrations of ANF were significantly lower than those inthe model group (Xia et al., 2019). Yang et al. (2019) found that quercetin restored the levels of superoxide dismutase, catalase, and glutathione in the liver of NAFLD mice. By activating the farnesoid X receptor 1 (FXR1)/TGR5 signaling pathway, quercetin eliminated lipid droplets and restored total cholesterol and triglyceride levels in HepG2 cells co-cultured with high d-glucose and free fatty acids. Wang et al. (2021) found that hyperoside can regulate bile acids (BAs) in the liver, reduce unconjugated BAs, and increase liver-conjugated BA levels. The expression of FXR in the liver is increased, leading to the promotion of free fatty acid β-oxidation.

### Regulate autophagy

Autophagy is a conserved self-digestion process that brings unnecessary or potentially dangerous cytoplasmic materials, such as damaged organelles and misfolded or unfolded proteins, to lysosomes for degradation. Lipid oxidation mainly occurs in the mitochondria, and oxidative stress produces a large amount of ROS, which leads to mitochondrial dysfunction and may inhibit autophagy because autophagy is generated in the mitochondria (Tang et al., 2017). Studies have shown that epigallocatechin-3-gallate (a flavonoid 3-alcohol phenolic compound) can increase the proliferation and autophagy of the liver in HFD-fed mice but reduce apoptosis. This may alleviate HFD-induced NAFLD by inhibiting apoptosis and promoting autophagy (Wu et al., 2021). Galangin is a flavonol and a curcumin derivative. Recent studies confirmed that galangin induces autophagy. Previous studies have reported that galangin mediates autophagy through the p53 pathway, and SIRT1 deacetylates LC3 in HepG2 cells (Zhang et al., 2021). Similarly, apigenin has been found to improve liver lipid deposition by activating mitochondrial autophagyto increase fatty acid β-oxidation and mitochondrial bioenergetics (Hsu et al., 2021).

### Anti-inflammatory effect

Oxidative stress-mediated inflammatory responses are an important pathological mechanism of NAFLD. When the level of oxidative stress increases, it can promote IL-6, IL-1β, and TNF-α expressionand induce liver injury (Xiao et al., 2018). The anti-inflammatory effect of flavonoids occurs mainly through the inhibition of the NF-κβ pathway (González et al., 2011). Flavonoids inhibit the phosphorylation of inhibitor of nuclear factor kappaB (IKB) and the inhibitor of nuclear factor kappaB kinase (IKK) complex (Kim et al., 2005) and the activity of regulatory enzymes, such asphospholipid oxygenase and protein tyrosine kinase (Manthey, 2009). Wang *et al.* found that the levels of IL-1β, IL-6, and TNF-α in the liver tissue of rats in the NAFLD model group were significantly increased, and total flavonoids of *Scutellaria baicalensis* could reduce these inflammatory factors, suggesting that total flavonoids in *Scutellaria baicalensis* could reduce the inflammatory response in the liver of rats in the NAFLD model group (Mengmeng et al., 2022). NO leads to highly destructive formation of peroxynitrite under oxidative stress conditions. Flavonoids inhibit inducible nitric oxide synthase (iNOS) expression and NO production (González-Gallego et al., 2010). In addition, flavonoids prevent the degeneration of the anti-inflammatory effects of the glucocorticoid cortisol. Oxidative stress worsens the anti-inflammatory effects of cortisol by eliminating these effects and creating cortisol resistance (Ruijters et al., 2014). Luteolin can significantly reduce a variety of inflammatory factors in NAFLD rats, which indicates that, in addition to its antioxidant effect, luteolin has also a very good anti-inflammatory effect (Abu-Elsaad and El-Karef, 2019). This suggests that NAFLD progression is often accompanied by inflammation and oxidative stress.

## Summary and prospect

The incidence of NAFLD increases each year, similar to clinical stress. Currently, NAFLD has an estimated annual medical and social cost of $292 billion (Younossi et al., 2016a). The different manifestations of NAFLD complicate the diagnosis, which ignores the true condition. The medical system is facing a severe challenge incombating this growing liver disease. Flavonoids have been proven to have very strong pharmacological activity and have excellent alleviating effects on NAFLD and NASH. Flavonoids may ameliorate NAFLD by regulating lipid metabolism, intestinal flora, and autophagy. Therefore, natural flavonoids have huge potential for the clinical development of NAFLD drugs in the future.

## Glosarry

**ALT**: alanine aminotransferase
**AMPK**: AMP-activated protein kinase
**ANF**: alpha-naphthoflavone
**ARE**: antioxidant response element
**AST**: aspartate aminotransferase
**BP**: broussonetiapyrifera
**C/EBP-α**: CCAAT/enhancer binding protein alpha
**ChREBP**: carbohydrate-responsive element-binding protein
**CPT1**: carnitine palmitoyltransferase 1A
**CYP7A1**: cytochrome P450 7A1
**DGAT2**: diacylglycerol acyltransferase 2
DNL: lysosomal DNA-ase
**E167K**: residue 167
**FA**: fatty acids
**FXR1**: farnesoid X receptor 1
**GCKR**: glucokinase regulator
**HO-1heme**: hemeoxygenase 1
**IKB**: inhibitor of nuclear factor kappaB
**IKK**: inhibitor of nuclear factor kappaB kinase
**IKKβ**: inhibitor of nuclear factor kappaB kinase beta
**IL-1β**: interleukin-1β
**IL-6**: interleukin 6
**iNOS**: inducible nitric oxide synthase
**IRE1α**: inositol-requiring enzyme 1 Alpha
**LPL**: lipoProtein lipase
**MBOAT7**: membrane bound o-acyltransferase domain-containing 7
**MDA**: malondialdehyde
**NAFLD**: non-alcoholic fatty liver disease
**NASH**: non-alcoholic steatohepatitis
**NASH-HCC**: non-alcoholic steatohepatitis-hepatocellular carcinoma
**NF-κβ**: nuclear factor kappa β
**NLRP3**: nucleotide-binding oligomerization domain, leucine-rich repeat and pyrin domain-containing 3
**Nrf2**: nuclear factor erythroid 2-related factor 2
**PC**: phosphatidylcholine
**PGC-1α**: peroxisome proliferator-activated receptor-gamma coactivator 1 alpha
**PI3K**: phosphatidylinositol-3-kinase
**PINK1**: putative kinase 1-mediated
**PNPLA3**: patatin-like phospholipase domain containing 3
**PPAR-α**: peroxisome proliferator-activated receptor alpha
**PPAR-γ**: peroxisome proliferator-activated receptor gamma
**ROS**: reactive oxygen species
**Sirt1**: sirtuin 1
**SOD**: speroxide Dismutase
**SREBP1c**: sterol regulatory binding protein-1c
**T2DM**: type 2 diabetes mellitus
**TAG**: triacylglycerol
**TC**: total cholesterol
**TFBP**: total flavonoids of broussonetia papyrifera
**TG**: triglyceride
**TM6SF2**: transmembrane 6 superfamily member 2
**TNF-α**: tumor necrosis factor-α
**TXA2**: thromboxane A2
**V-ATPase**: vacuolar proton ATPase
**VLDL**: very low density lipoprotein
**XBP1s**: X-box binding protein 1 spliced
